# Supplementary material for: Single‐Cell and Spatial Transcriptomics Unveil Key Regulators Governing Cell Differentiation for Schistosoma japonicum Sexual Development
Source: Adv Sci (Weinh). 2026 Jul 11;13(53):e76329. doi: 10.1002/advs.76329 (PMC13355927; doi:10.1002/advs.76329)
Supplement: Supplementary file 2 [file ADVS-13-e76329-s004.docx]

**Supplementary information:**

**Figure S1.** Identification of cell populations in *S. japonicum*. (**A**) Marker genes in different cell types. (**B**) UMAP showing different cell types. (**C**) Validation of anatomical localizations of representatively identified tissue cells. Red dashed lines on UMAPs highlight the relevant cell cluster. Data are representative results of 20-30 investigated worms. Scale bars = 200 µm.

Figure S2. Spatial transcriptomic analyses of *S. japonicum*. (A) Spatial transcriptomic marker genes in different cell types. (B) Spatial transcriptomic analyses of representative cell types within paired adult parasites.

Figure S3. The heterogeneity of neuron cells. Heatmap of neuronal maker genes (A) and A dot-plot summarizing the genes highly expressing in each cluster (B). (C) Double FISH analysis indicated the combination of neuronal cluster-specific markers for neuronal cell population. EWB00_008401, Synaptic vesicle membrane protein VAT-1 isoform 1; EWB00_008189, Calcium uptake protein 2 isoform 2; EWB00_004539, Tyrosine-protein kinase.

Figure S4. The heterogeneity of muscle cells. (A) Heatmap of muscle maker genes and (B) A dot-plot summarizing the genes highly expressing in each cluster.

Figure S5. The heterogeneity of tegument cells. (A) Heatmap of tegument maker genes and (B) A dot-plot summarizing the genes highly expressing in each cluster.

Figure S6 The heterogeneity of parenchyma cells. (A) Heatmap of parenchyma maker genes and (B) A dot-plot summarizing the genes highly expressing in each cluster.

Figure S7 Transcriptomic dynamics during *S. japonicum* sexual maturation. (A) Gene expression correlations between different samples. (B) UMAP showing the signature score from clustered genes in different cell types between males and females. M, male; F, female.

**Figure S8** EdU staining analysis of cell proliferation in the vitellocytes of *Zeb2* KD females. Data are shown a representative image from 8-10 females. Numbers given indicate the fraction of worms that were similar with respect to the phenotype in relation to the total number of worms examined. Scale bars = 60 μm.

**Figure S9** Ovarian proliferation remained unaffected in *Zfp* and *Fbp3* KD females. Data are shown a representative image from 17-21 females. Numbers given indicate the fraction of worms that were similar with respect to the phenotype in relation to the total number of worms examined. Scale bars = 100 μm.

**Figure S10** EdU staining analysis of cell proliferation in the vitellocytes of *H2a* KD females. Data are shown a representative image from 18-21 females. Numbers given indicate the fraction of worms that were similar with respect to the phenotype in relation to the total number of worms examined. Scale bars = 50 μm.

**Figure S11** Comparison of scRNA-seq data between *S. japonicum* and *S. mansoni.* (**A**) Clustering of integrated scRNA-seq data for *S. japonicum* and *S. mansoni*. (**B**) Unsupervised clustering and source of origin in each cluster. (**C**) Cross-species correlation analysis for identified cell types. (**D**) Dot plot showing cell type expression of identified key genes. The three modules represent shared upregulated genes in both GSC and S1 (Shared_Up, 60%), and lineage-specific upregulated genes (GSC-only and S1-only, 40%) corresponding to Fig 8D and Supplementary Table 9. (**E**) Screenshots showing the query results from the schistosome expression database.

**Supplementary Tables**

**Table S1** The list of cluster maker genes in each cell population.

**Table S2** The list of marker genes for different cell types from spatial transcriptomics.

**Table S3** The list of marker genes for Figure S2B.

**Table S4** The list of identified genes in different clusters by fuzzy clustering analysis and their GO enrichment results.

**Table S5** The list of differently expressed genes between D20 and D16.

**Table S6** The list of identified transcription factors in *S. japonicum.*

**Table S7** The list of differentially expressed genes between F26 and M26.

**Table S8** The list of maker genes of *Ago2^+^* cell populations in D16/D20/D26.

**Table S9** The lists of differentially expressed genes of S1 and germinal stem cells between *S. mansoni* and *S. japonicum*.

**Table S10** The list of primers used in the present study.
