# Supplementary material for: Single‐Cell and Spatial Transcriptomics Unveil Key Regulators Governing Cell Differentiation for Schistosoma japonicum Sexual Development
Source: Adv Sci (Weinh). 2026 Jul 11:e76329. Online ahead of print. doi: 10.1002/advs.76329 (PMC13355927; doi:10.1002/advs.76329)
Supplement: Supplementary file 2 — Supporting File 2: advs76329‐sup‐0002‐Fig S1‐S11.pdf. [file ADVS-9999-e76329-s010.pdf]

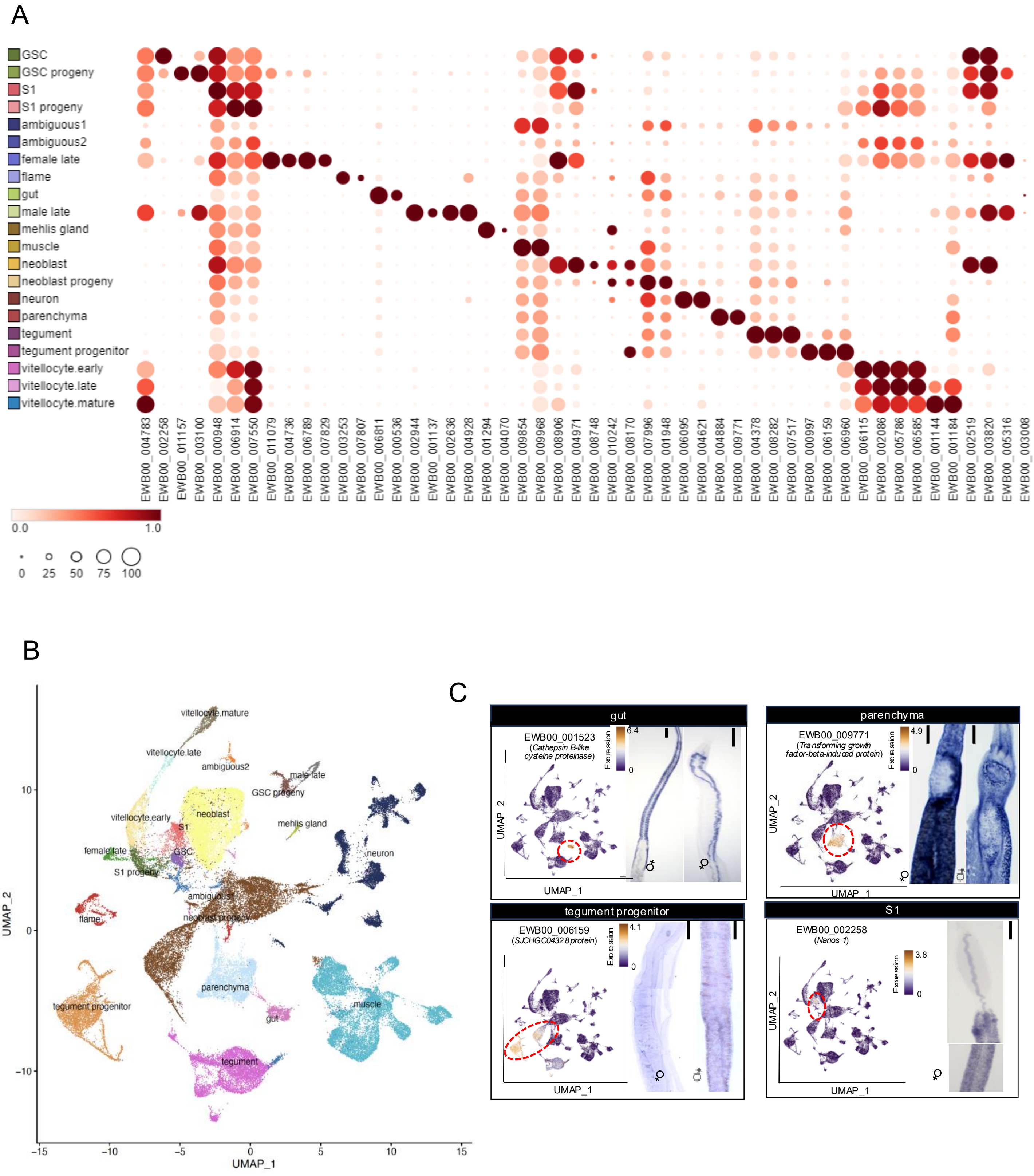

A
GSC
GSC progeny
S1
S1 progeny
ambiguous1
ambiguous2
female late
flame
gut
male late
mehlis gland
muscle
neoblast
neoblast progeny
neuron
parenchyma
tegument
tegument progenitor
vitellocyte.early
vitellocyte.late
vitellocyte.mature
EWB00_004783
EWB00_002258
EWB00_011157
EWB00_003100
EWB00_000948
EWB00_006914
EWB00_007550
EWB00_011079
EWB00_004736
EWB00_006789
EWB00_007829
EWB00_003253
EWB00_007807
EWB00_006811
EWB00_000536
EWB00_002944
EWB00_001137
EWB00_002636
EWB00_004928
EWB00_001294
EWB00_004070
EWB00_009854
EWB00_009968
EWB00_008906
EWB00_004971
EWB00_008748
EWB00_010242
EWB00_008170
EWB00_007996
EWB00_001948
EWB00_006095
EWB00_004621
EWB00_004884
EWB00_009771
EWB00_004378
EWB00_008282
EWB00_007517
EWB00_000997
EWB00_006159
EWB00_006960
EWB00_006115
EWB00_002086
EWB00_005786
EWB00_006585
EWB00_001144
EWB00_001184
EWB00_002519
EWB00_003820
EWB00_005316
EWB00_003008
0.0
1.0
0
25
50
75
100
B
vitellocyte.mature
vitellocyte.late
ambiguous2
male late
GSC progeny
neoblast
mehlis gland
vitellocyte.early
S1
GSC
female late
S1 progeny
neuron
ambiguous1
neoblast progeny
flame
parenchyma
gut
muscle
tegument progenitor
tegument
UMAP_2
UMAP_1
10
0
−10
−15
−10
−5
0
5
10
15
C
gut
EWB00_001523
(Cathepsin B-like cysteine proteinase)
6.4
0
Expression
UMAP_2
UMAP_1
parenchyma
EWB00_009771
(Transforming growth factor-beta-induced protein)
4.9
0
tegument progenitor
EWB00_006159
(SJCHGC04328 protein)
4.1
0
S1
EWB00_002258
(Nanos 1)
3.8
0

Figure S1

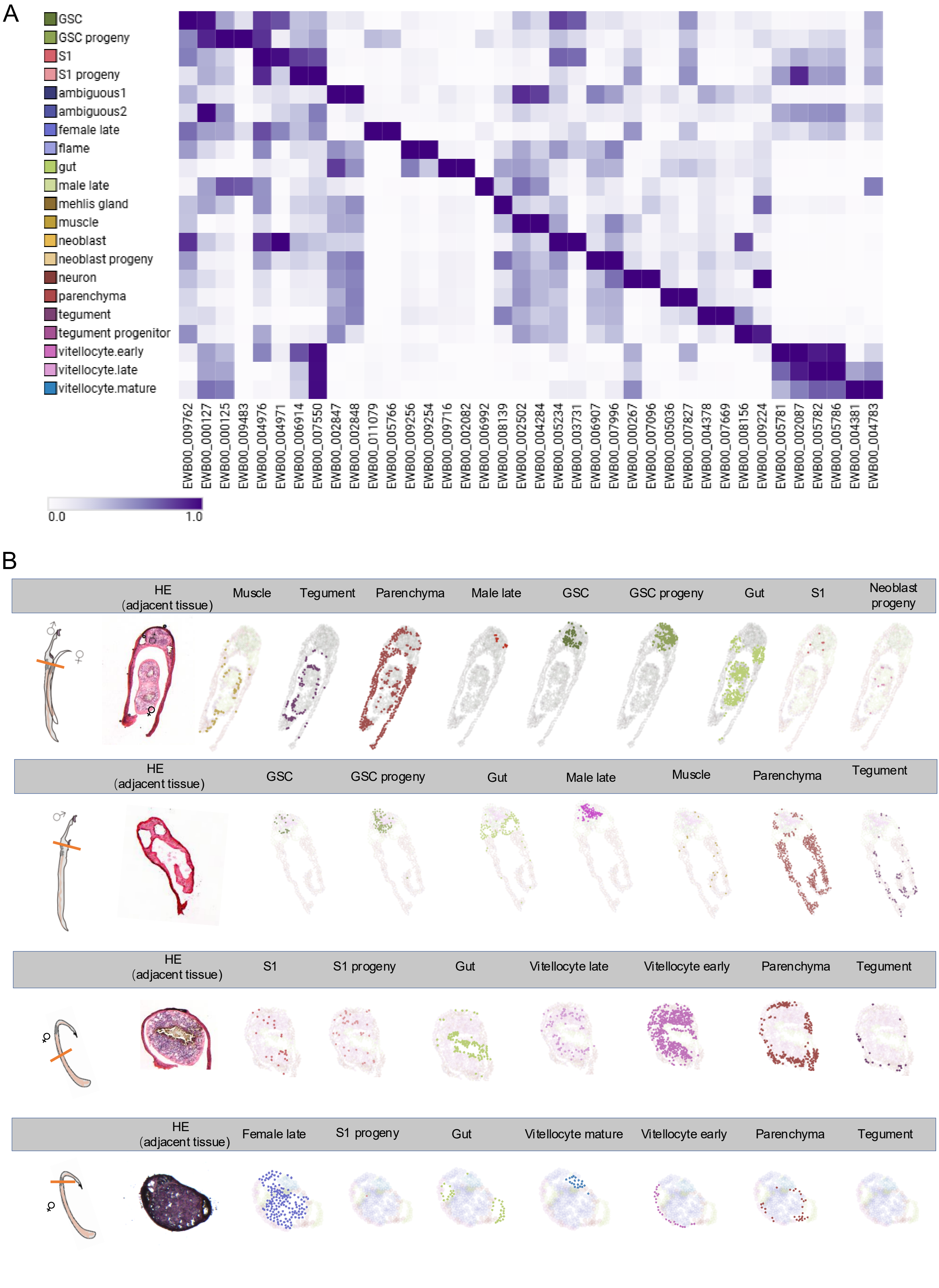
A
GSC
GSC progeny
S1
S1 progeny
ambiguous1
ambiguous2
female late
flame
gut
male late
mehlis gland
muscle
neoblast
neoblast progeny
neuron
parenchyma
tegument
tegument progenitor
vitellocyte.early
vitellocyte.late
vitellocyte.mature
EWB00_009762
EWB00_000127
EWB00_000125
EWB00_009483
EWB00_004976
EWB00_004971
EWB00_006914
EWB00_007550
EWB00_002847
EWB00_002848
EWB00_011079
EWB00_005766
EWB00_009256
EWB00_009254
EWB00_009716
EWB00_002082
EWB00_006992
EWB00_008139
EWB00_002502
EWB00_004284
EWB00_005234
EWB00_003731
EWB00_006907
EWB00_007996
EWB00_000267
EWB00_007096
EWB00_005036
EWB00_007827
EWB00_004378
EWB00_007669
EWB00_008156
EWB00_009224
EWB00_005781
EWB00_002087
EWB00_005782
EWB00_005786
EWB00_004381
EWB00_004783
0.0
1.0
B
HE (adjacent tissue)
Muscle
Tegument
Parenchyma
Male late
GSC
GSC progeny
Gut
S1
Neoblast progeny
HE (adjacent tissue)
GSC
GSC progeny
Gut
Male late
Muscle
Parenchyma
Tegument
HE (adjacent tissue)
S1
S1 progeny
Gut
Vitellocyte late
Vitellocyte early
Parenchyma
Tegument
HE (adjacent tissue)
Female late
S1 progeny
Gut
Vitellocyte mature
Vitellocyte early
Parenchyma
Tegument

Figure S2

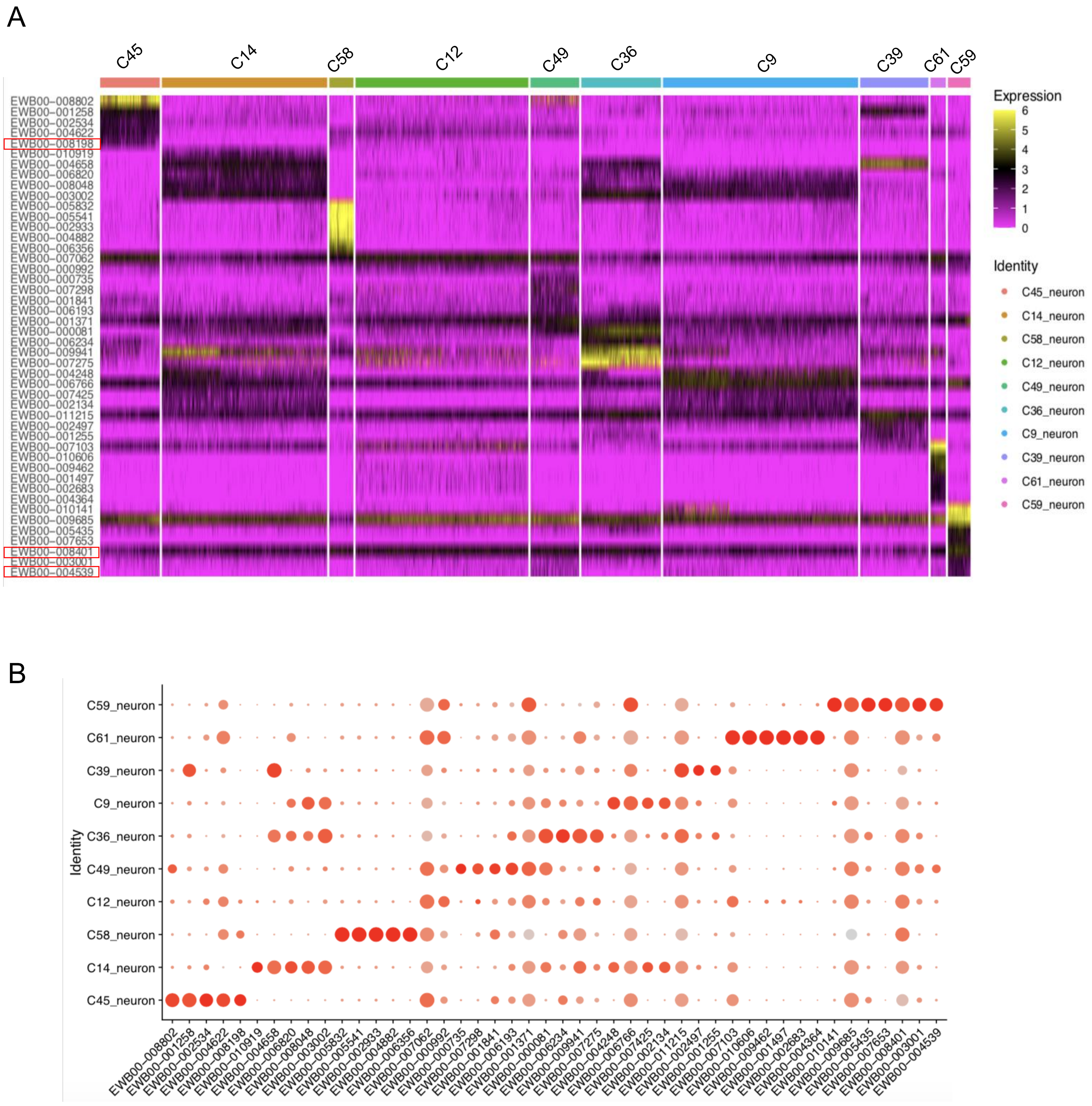
A
C45
C14
C58
C12
C49
C36
C9
C39
C61
C59
Expression
Identity
C45_neuron
C14_neuron
C58_neuron
C12_neuron
C49_neuron
C36_neuron
C9_neuron
C39_neuron
C61_neuron
C59_neuron
B
Identity

Figure S3

C

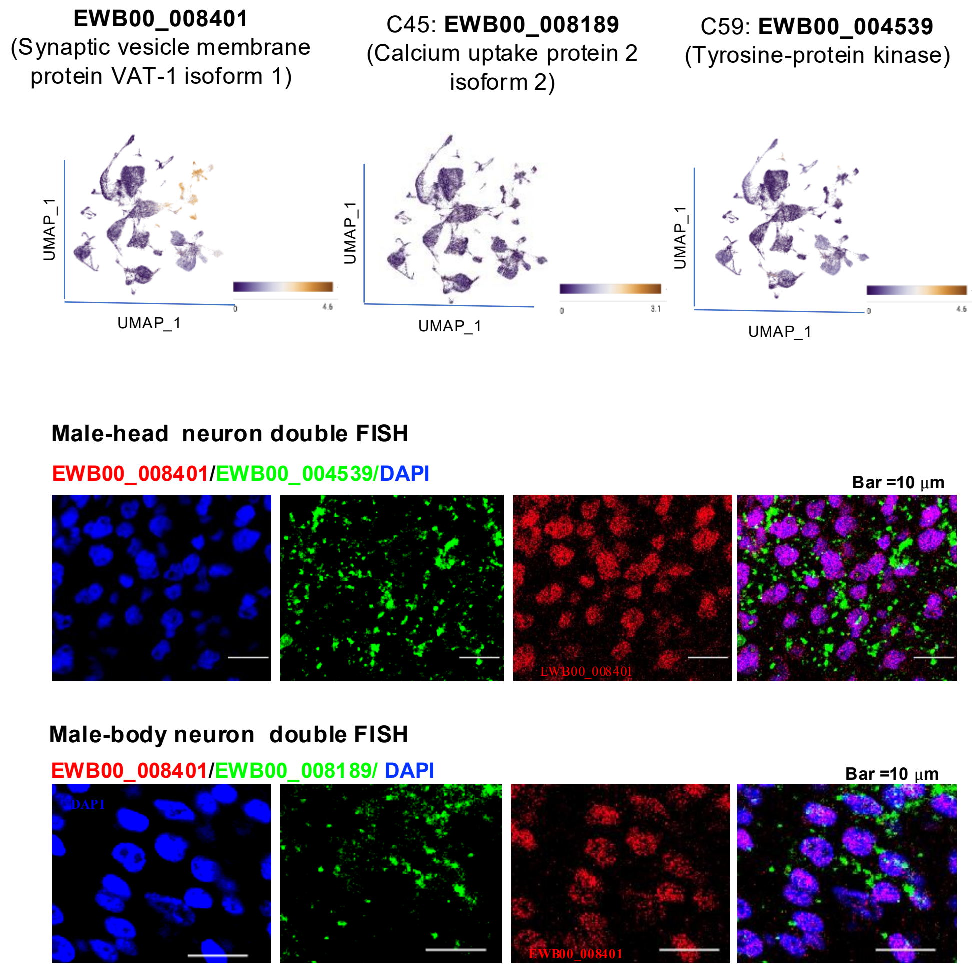

EWB00_008401
(Synaptic vesicle membrane protein VAT-1 isoform 1)
C45: EWB00_008189
(Calcium uptake protein 2 isoform 2)
C59: EWB00_004539
(Tyrosine-protein kinase)
UMAP_1
UMAP_1
UMAP_1
UMAP_1
UMAP_1
UMAP_1
Male-head neuron double FISH
EWB00_008401/EWB00_004539/DAPI
Bar =10 µm
EWB00_008401
Male-body neuron double FISH
EWB00_008401/EWB00_008189/ DAPI
Bar =10 µm
DAPI
EWB00_008401

Figure S3

A

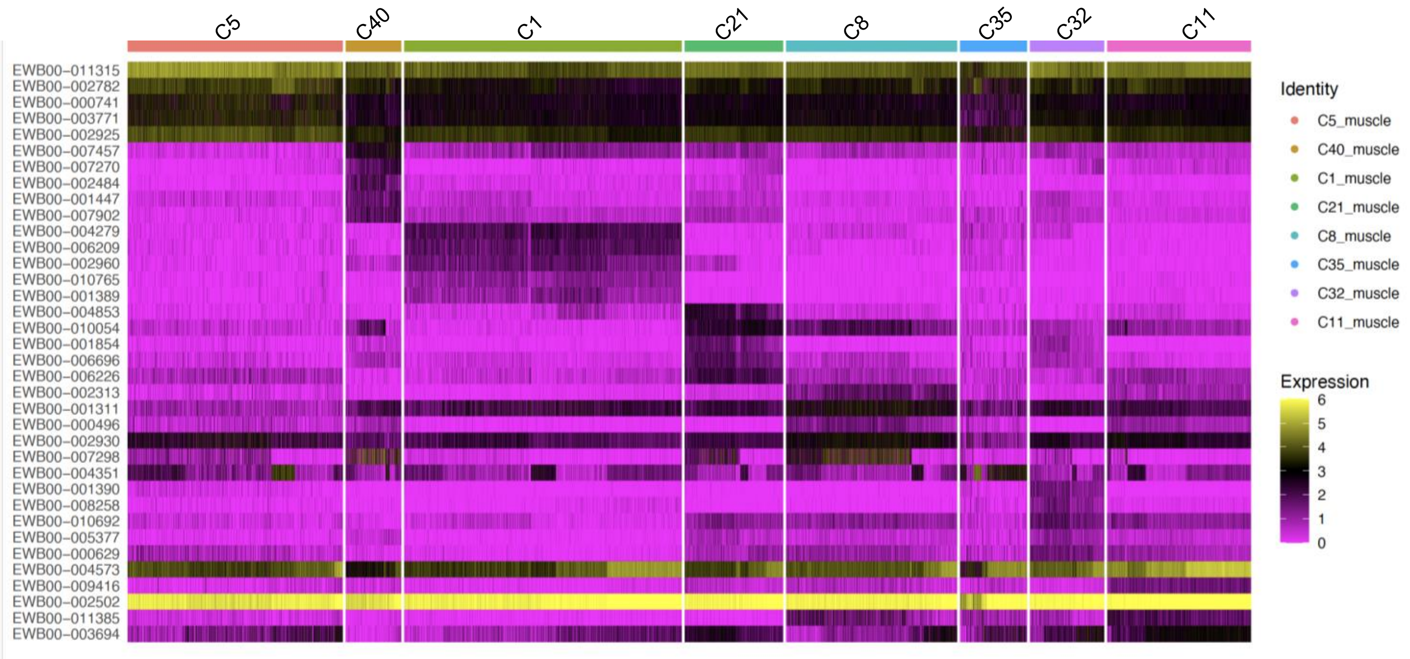
C5
C40
C1
C21
C8
C35
C32
C11
EWB00-011315
EWB00-002782
EWB00-000741
EWB00-003771
EWB00-002925
EWB00-007457
EWB00-007270
EWB00-002484
EWB00-001447
EWB00-007902
EWB00-004279
EWB00-006209
EWB00-002960
EWB00-010765
EWB00-001389
EWB00-004853
EWB00-010054
EWB00-001854
EWB00-006696
EWB00-006226
EWB00-002313
EWB00-001311
EWB00-000496
EWB00-002930
EWB00-007298
EWB00-004351
EWB00-001390
EWB00-008258
EWB00-010692
EWB00-005377
EWB00-000629
EWB00-004573
EWB00-009416
EWB00-002502
EWB00-011385
EWB00-003694
Identity
C5_muscle
C40_muscle
C1_muscle
C21_muscle
C8_muscle
C35_muscle
C32_muscle
C11_muscle
Expression
6
5
4
3
2
1
0

B

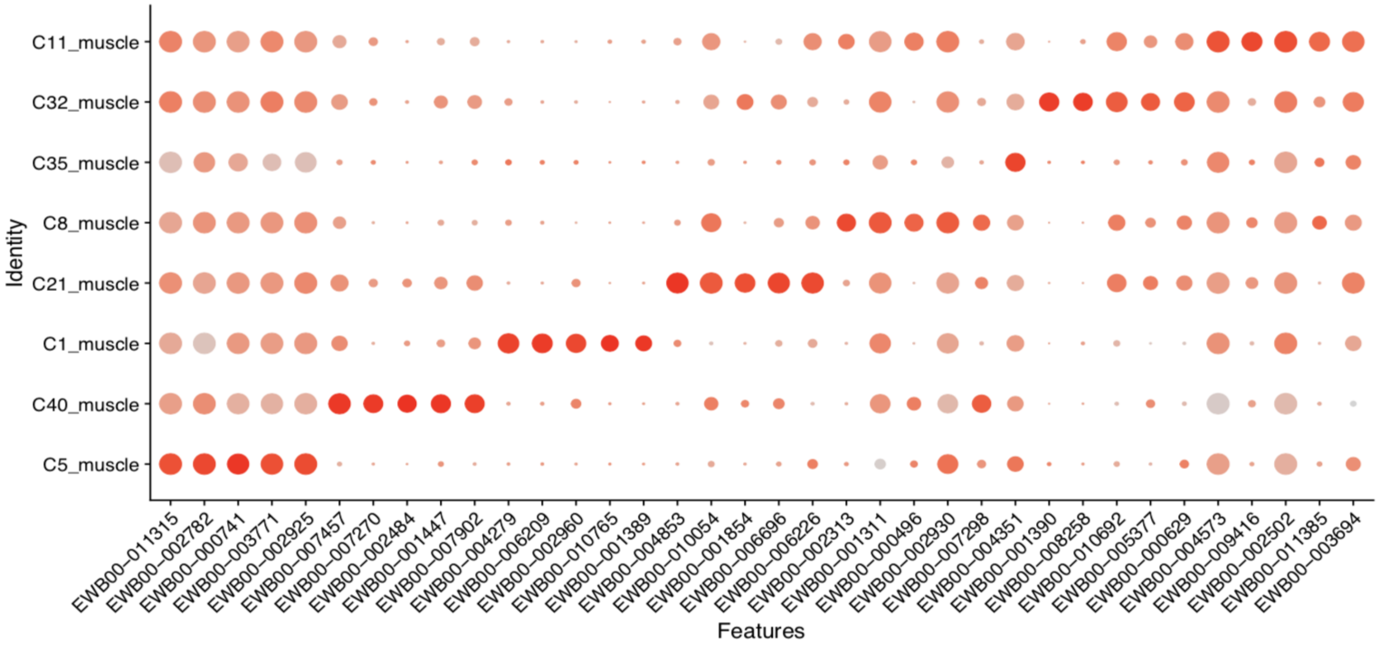
C11_muscle
C32_muscle
C35_muscle
C8_muscle
C21_muscle
C1_muscle
C40_muscle
C5_muscle
Identity
EWB00-011315
EWB00-002782
EWB00-000741
EWB00-003771
EWB00-002925
EWB00-007457
EWB00-007270
EWB00-002484
EWB00-001447
EWB00-007902
EWB00-004279
EWB00-006209
EWB00-002960
EWB00-010765
EWB00-001389
EWB00-004853
EWB00-010054
EWB00-001854
EWB00-006696
EWB00-006226
EWB00-002313
EWB00-001311
EWB00-000496
EWB00-002930
EWB00-007298
EWB00-004351
EWB00-001390
EWB00-008258
EWB00-010692
EWB00-005377
EWB00-000629
EWB00-004573
EWB00-009416
EWB00-002502
EWB00-011385
EWB00-003694
Features

Figure S4

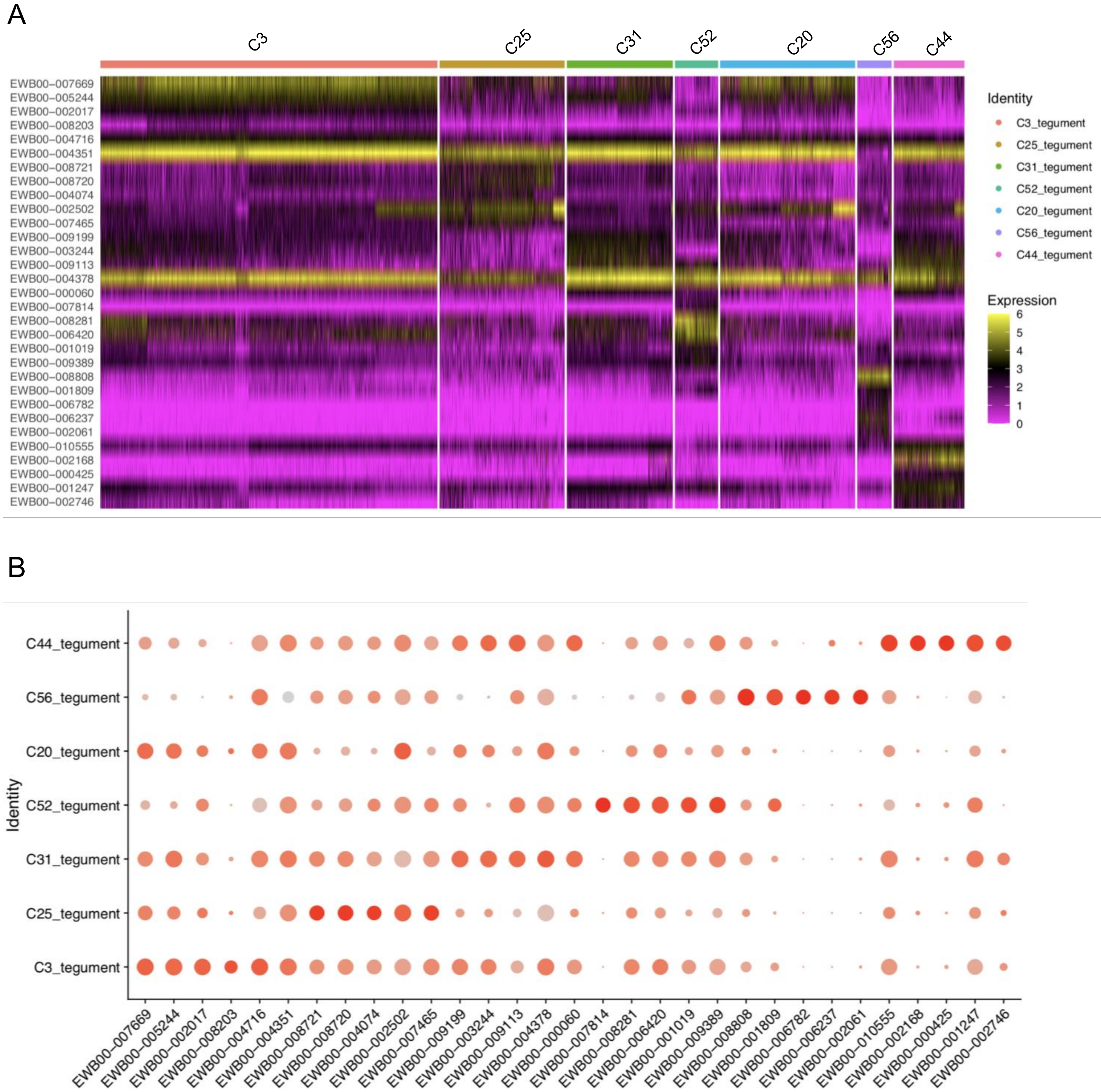

A
C3
C25
C31
C52
C20
C56
C44
EWB00–007669
EWB00–005244
EWB00–002017
EWB00–008203
EWB00–004716
EWB00–004351
EWB00–008721
EWB00–008720
EWB00–004074
EWB00–002502
EWB00–007465
EWB00–009199
EWB00–003244
EWB00–009113
EWB00–004378
EWB00–000060
EWB00–007814
EWB00–008281
EWB00–006420
EWB00–001019
EWB00–009389
EWB00–008808
EWB00–001809
EWB00–006782
EWB00–006237
EWB00–002061
EWB00–010555
EWB00–002168
EWB00–000425
EWB00–001247
EWB00–002746
Identity
C3_tegument
C25_tegument
C31_tegument
C52_tegument
C20_tegument
C56_tegument
C44_tegument
Expression
6
5
4
3
2
1
0
B
Identity
C44_tegument
C56_tegument
C20_tegument
C52_tegument
C31_tegument
C25_tegument
C3_tegument

Figure S5

A

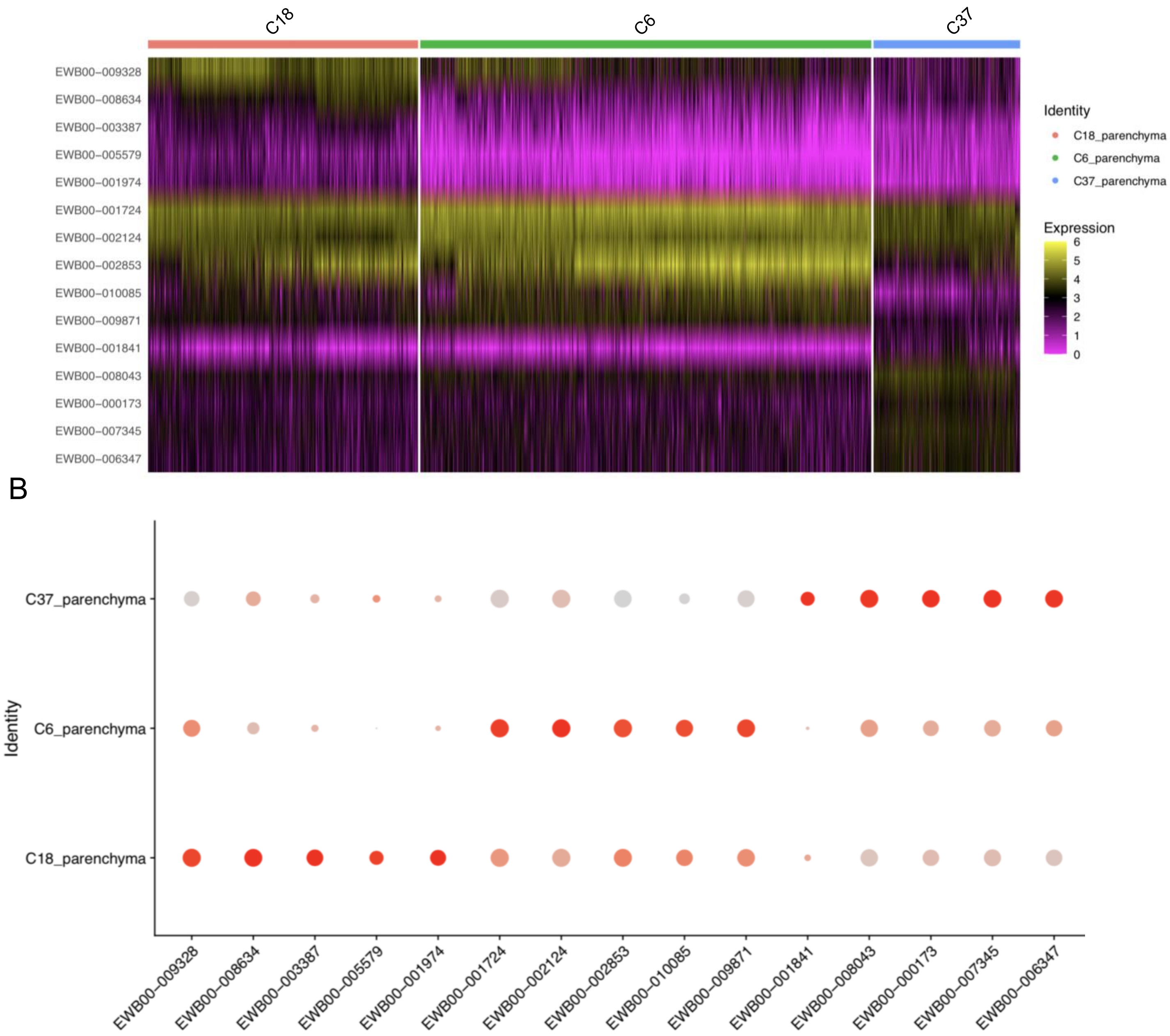

C18
C6
C37
EWB00–009328
EWB00–008634
EWB00–003387
EWB00–005579
EWB00–001974
EWB00–001724
EWB00–002124
EWB00–002853
EWB00–010085
EWB00–009871
EWB00–001841
EWB00–008043
EWB00–000173
EWB00–007345
EWB00–006347
Identity
C18_parenchyma
C6_parenchyma
C37_parenchyma
Expression
6
5
4
3
2
1
0
B
C37_parenchyma
C6_parenchyma
C18_parenchyma
Identity

Figure S6

A

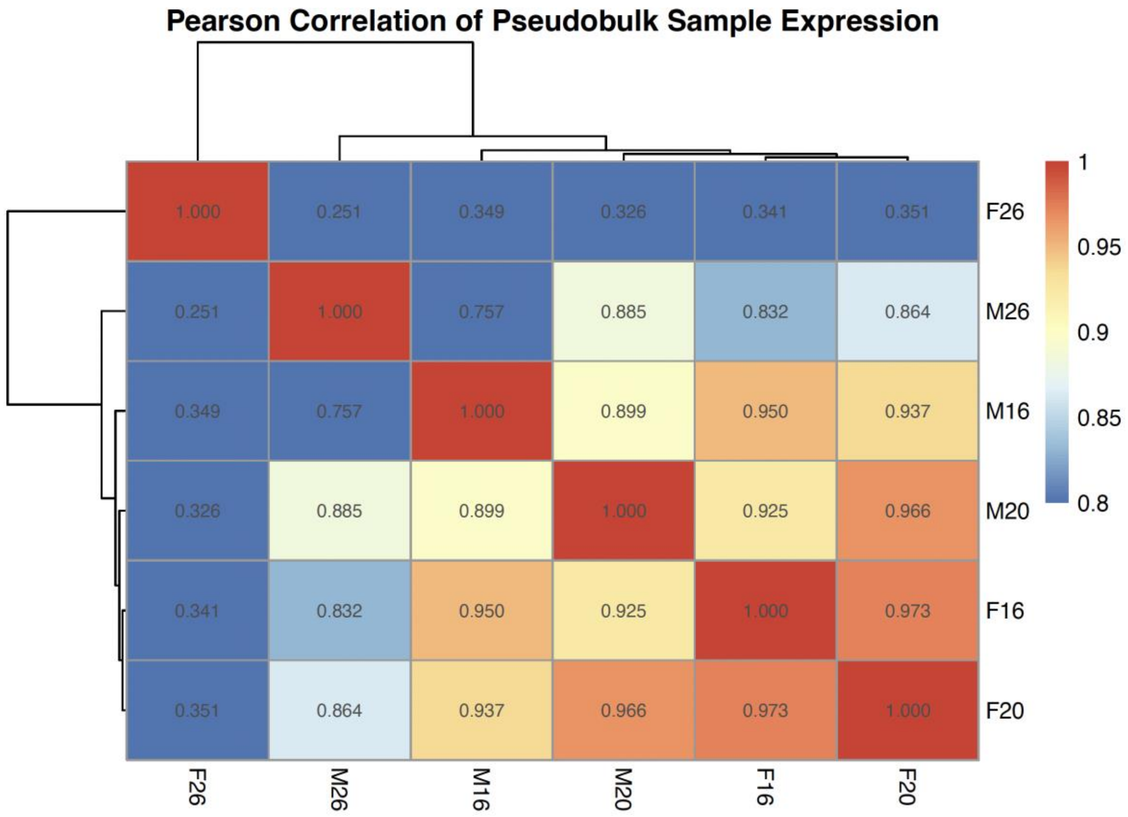
Pearson Correlation of Pseudobulk Sample Expression
F26
M26
M16
M20
F16
F20
1.000
0.251
0.349
0.326
0.341
0.351
0.757
0.885
0.832
0.864
0.899
0.950
0.937
0.925
0.966
0.973
1
0.95
0.9
0.85
0.8

Figure S7

B

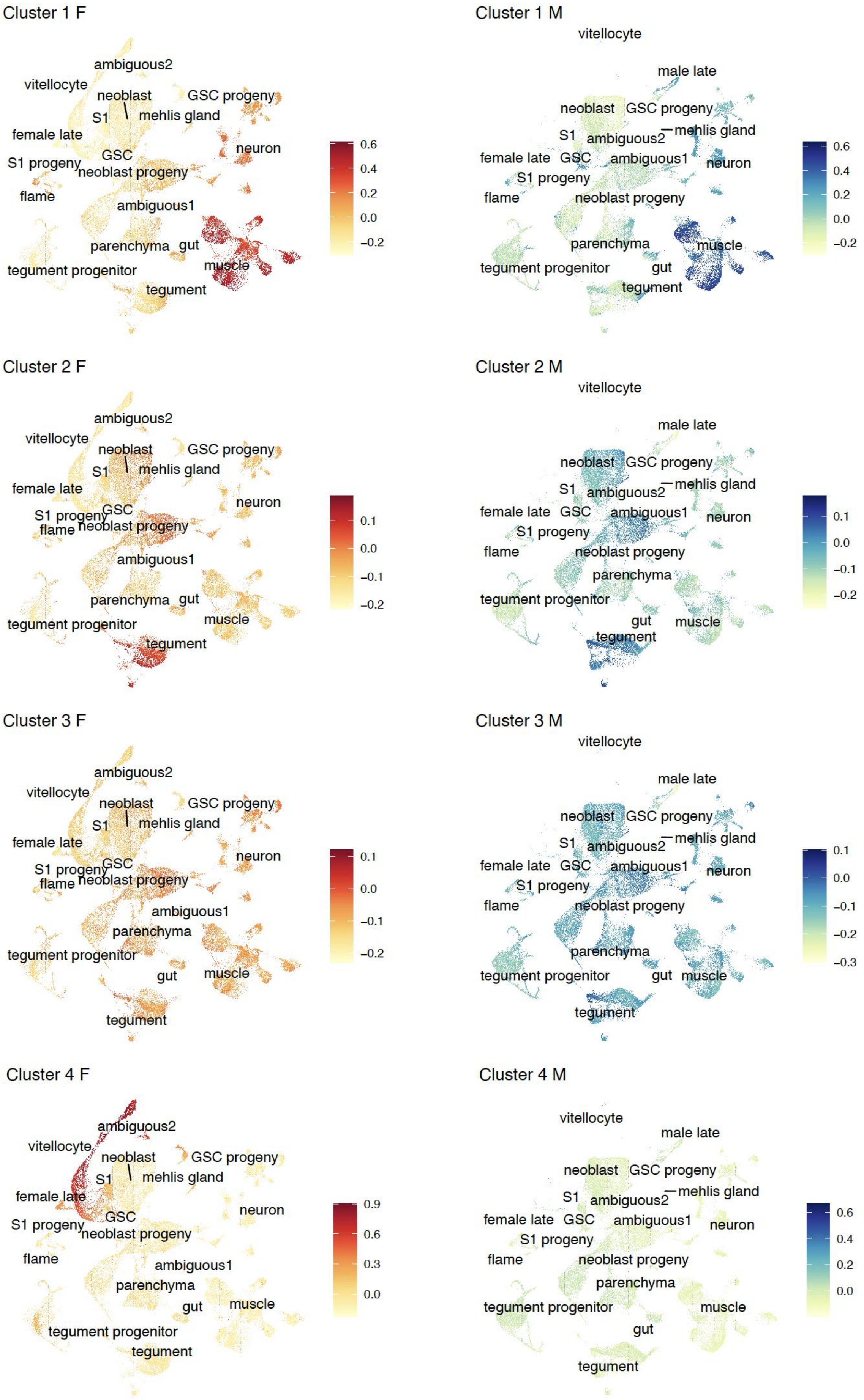
Cluster 1 F
ambiguous2
vitellocyte
neoblast
GSC progeny
S1
mehlis gland
female late
neuron
S1 progeny
GSC
neoblast progeny
flame
ambiguous1
parenchyma
gut
tegument progenitor
muscle
tegument
0.6
0.4
0.2
0.0
−0.2
Cluster 1 M
vitellocyte
male late
neoblast
GSC progeny
S1
ambiguous2
mehlis gland
female late
GSC
ambiguous1
neuron
S1 progeny
flame
neoblast progeny
parenchyma
muscle
tegument progenitor
gut
tegument
0.6
0.4
0.2
0.0
−0.2
Cluster 2 F
0.1
0.0
−0.1
−0.2
Cluster 2 M
0.1
0.0
−0.1
−0.2
Cluster 3 F
0.1
0.0
−0.1
−0.2
Cluster 3 M
0.1
0.0
−0.1
−0.2
−0.3
Cluster 4 F
0.9
0.6
0.3
0.0
Cluster 4 M
0.6
0.4
0.2
0.0

Figure S7

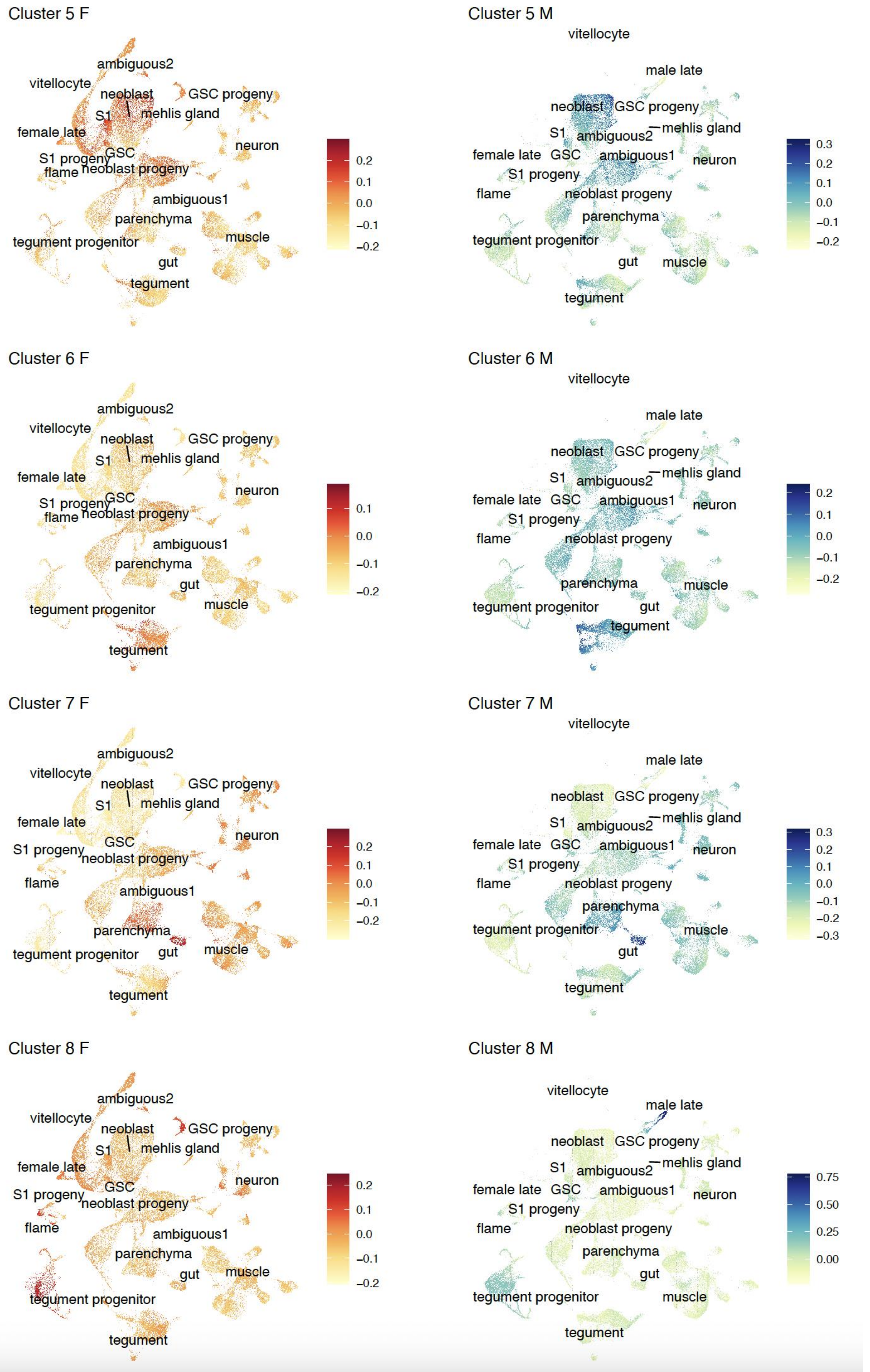

Cluster 5 F
ambiguous2
vitellocyte
neoblast
GSC progeny
S1
mehlis gland
female late
neuron
S1 progeny
GSC
flame
neoblast progeny
ambiguous1
parenchyma
tegument progenitor
muscle
gut
tegument
0.2
0.1
0.0
−0.1
−0.2
Cluster 5 M
vitellocyte
male late
neoblast
GSC progeny
S1
ambiguous2
mehlis gland
female late
GSC
ambiguous1
neuron
S1 progeny
flame
neoblast progeny
parenchyma
tegument progenitor
gut
muscle
tegument
0.3
0.2
0.1
0.0
−0.1
−0.2
Cluster 6 F
ambiguous2
vitellocyte
neoblast
GSC progeny
S1
mehlis gland
female late
neuron
S1 progeny
GSC
flame
neoblast progeny
ambiguous1
parenchyma
gut
tegument progenitor
muscle
tegument
0.1
0.0
−0.1
−0.2
Cluster 6 M
vitellocyte
male late
neoblast
GSC progeny
S1
ambiguous2
mehlis gland
female late
GSC
ambiguous1
neuron
S1 progeny
flame
neoblast progeny
parenchyma
muscle
tegument progenitor
gut
tegument
0.2
0.1
0.0
−0.1
−0.2
Cluster 7 F
ambiguous2
vitellocyte
neoblast
GSC progeny
S1
mehlis gland
female late
neuron
S1 progeny
GSC
neoblast progeny
flame
ambiguous1
parenchyma
tegument progenitor
gut
muscle
tegument
0.2
0.1
0.0
−0.1
−0.2
Cluster 7 M
vitellocyte
male late
neoblast
GSC progeny
S1
ambiguous2
mehlis gland
female late
GSC
ambiguous1
neuron
S1 progeny
flame
neoblast progeny
parenchyma
tegument progenitor
muscle
gut
tegument
0.3
0.2
0.1
0.0
−0.1
−0.2
−0.3
Cluster 8 F
ambiguous2
vitellocyte
neoblast
GSC progeny
S1
mehlis gland
female late
neuron
S1 progeny
GSC
neoblast progeny
flame
ambiguous1
parenchyma
gut
muscle
tegument progenitor
tegument
0.2
0.1
0.0
−0.1
−0.2
Cluster 8 M
vitellocyte
male late
neoblast
GSC progeny
S1
ambiguous2
mehlis gland
female late
GSC
ambiguous1
neuron
S1 progeny
flame
neoblast progeny
parenchyma
gut
tegument progenitor
muscle
tegument
0.75
0.50
0.25
0.00

Figure S7

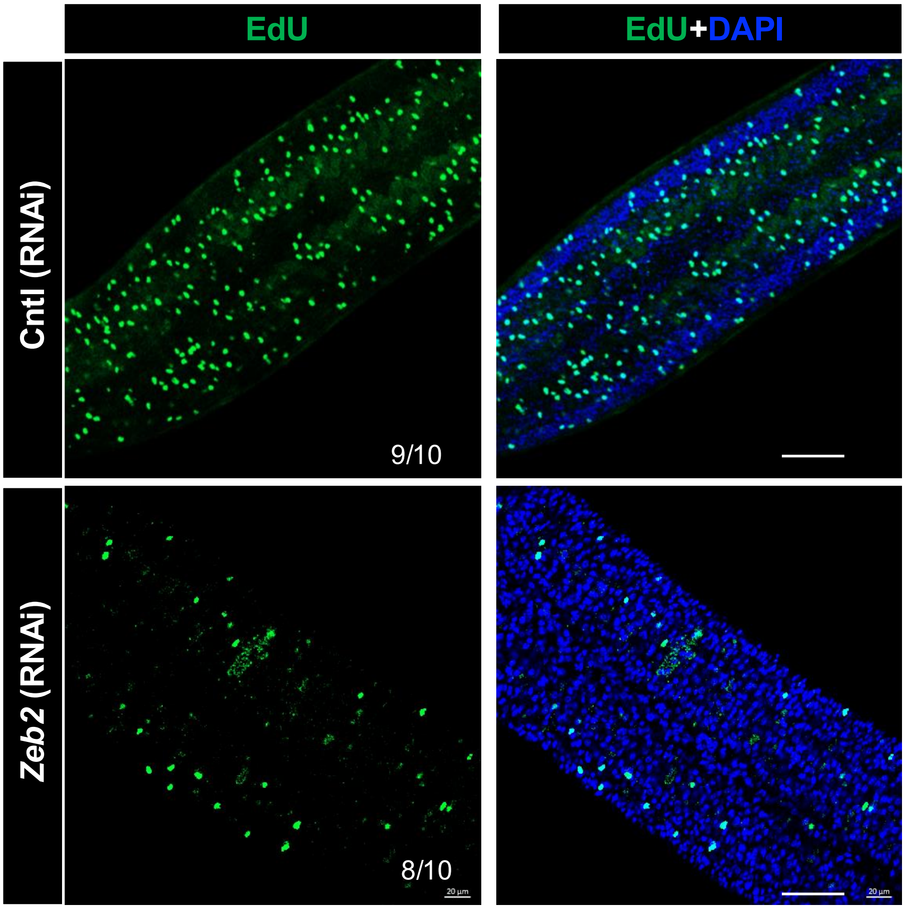

Figure S8

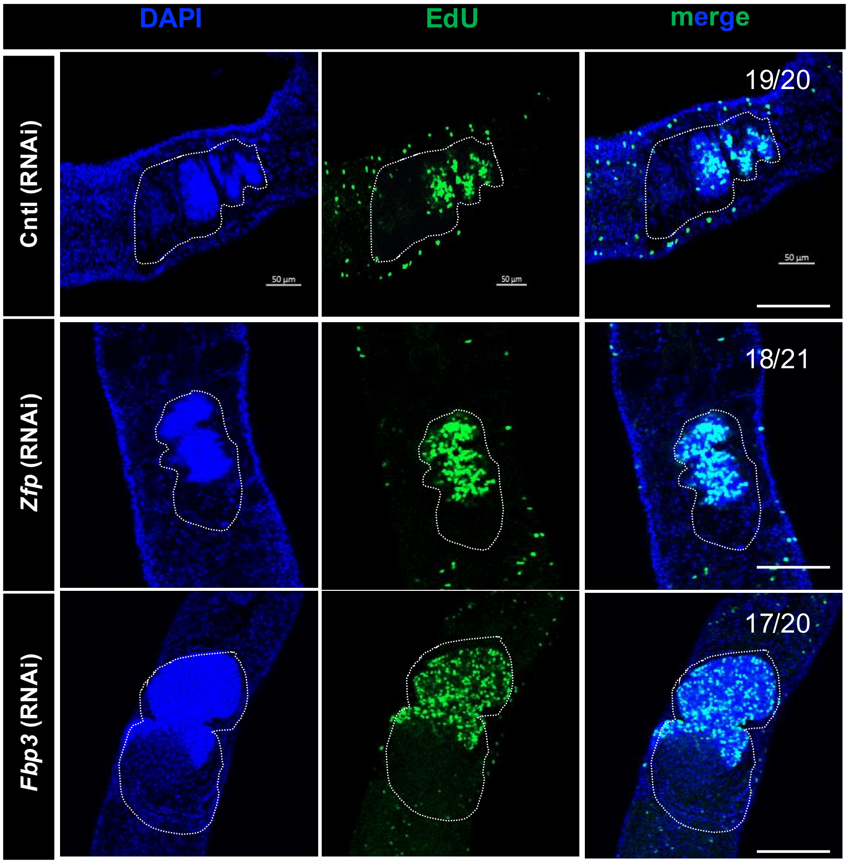
DAPI
EdU
merge
Cntl (RNAi)
19/20
50 µm
50 µm
50 µm
Zfp (RNAi)
18/21
Fbp3 (RNAi)
17/20

Figure S9

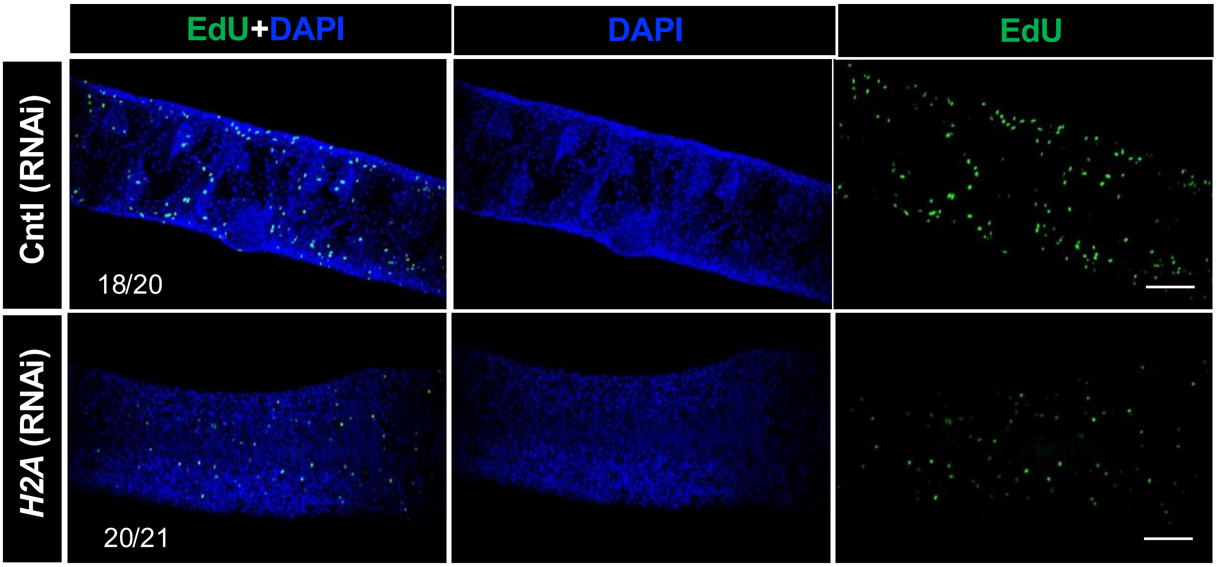

Figure S10

A

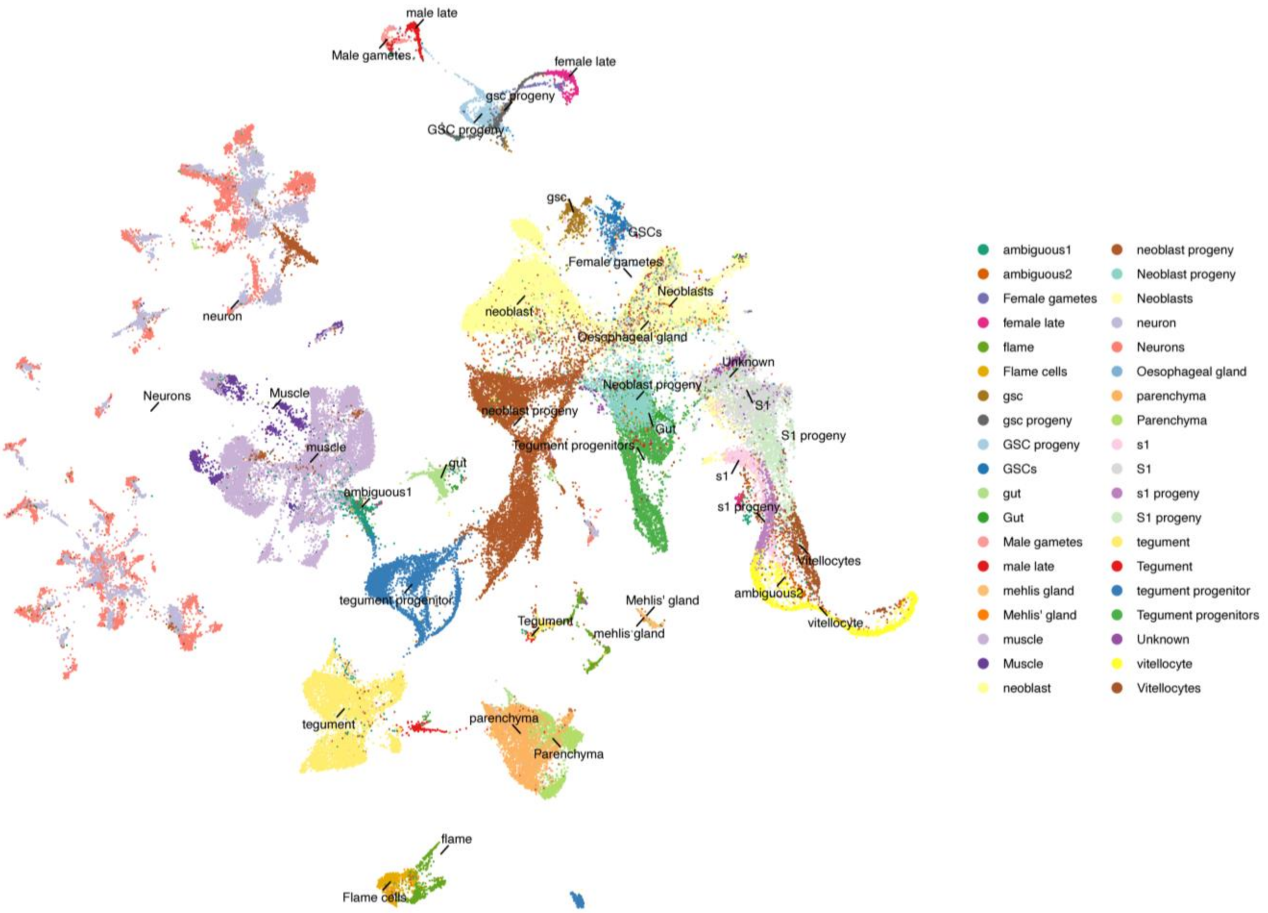

B

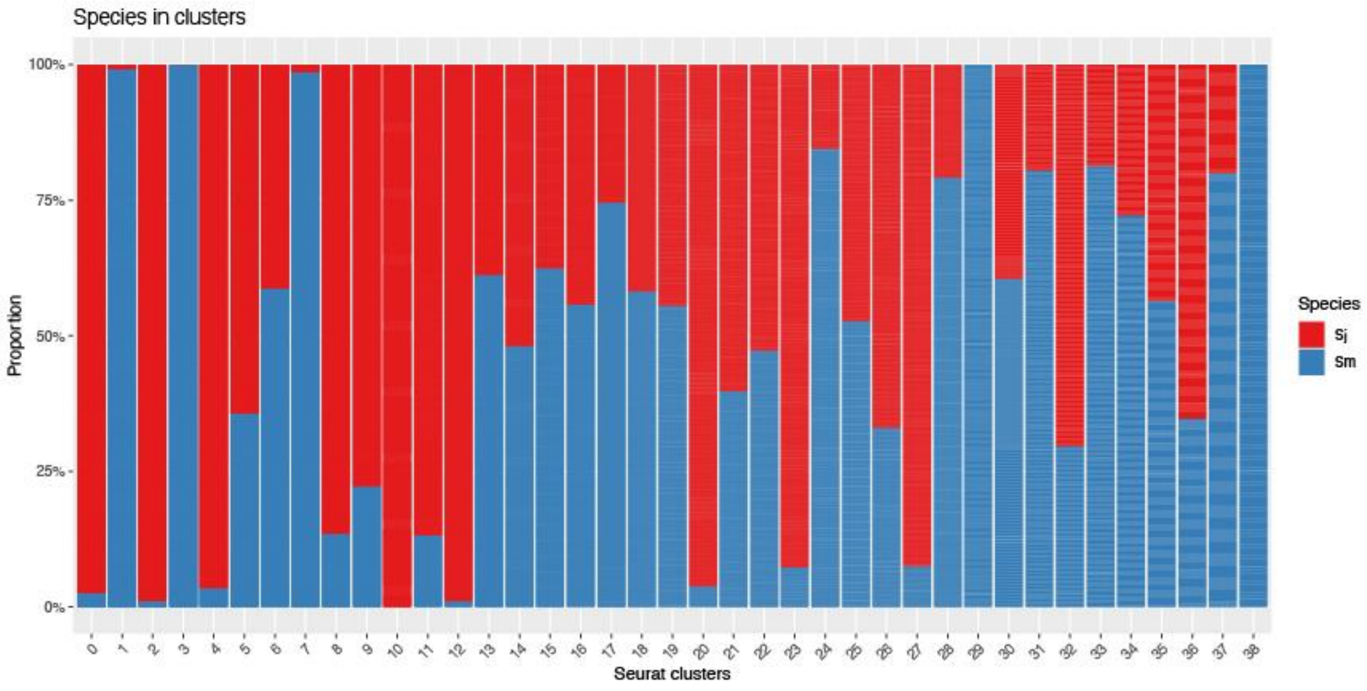

Figure S11

C

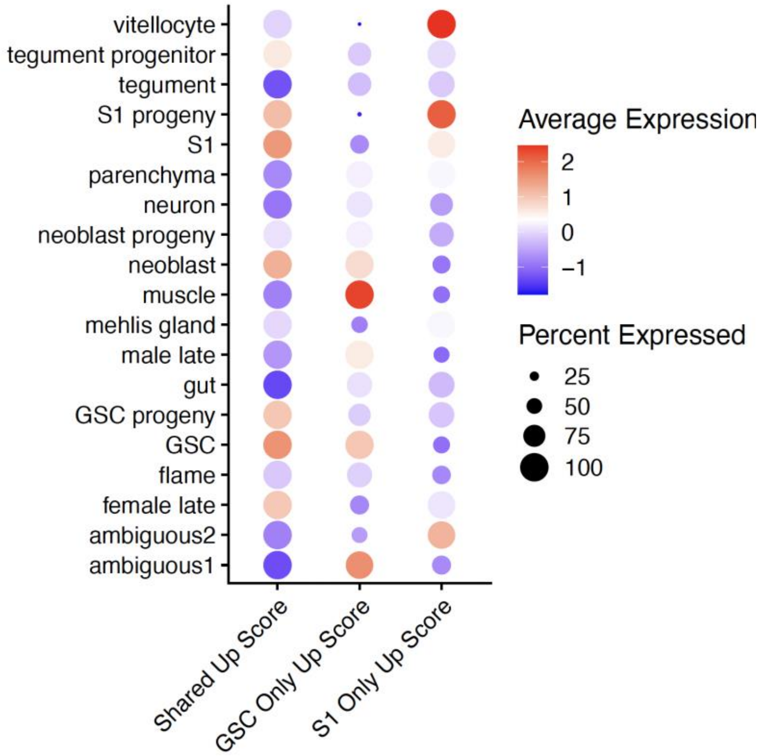

D

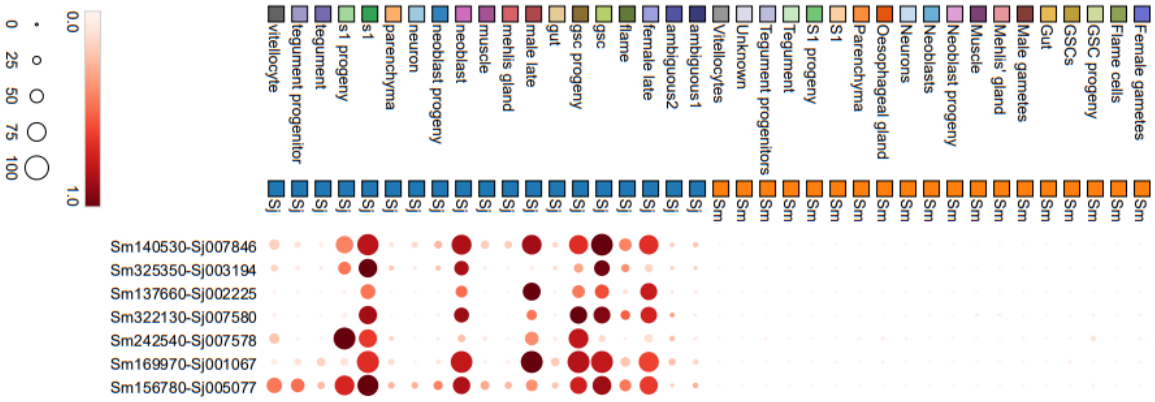

Figure S11

E

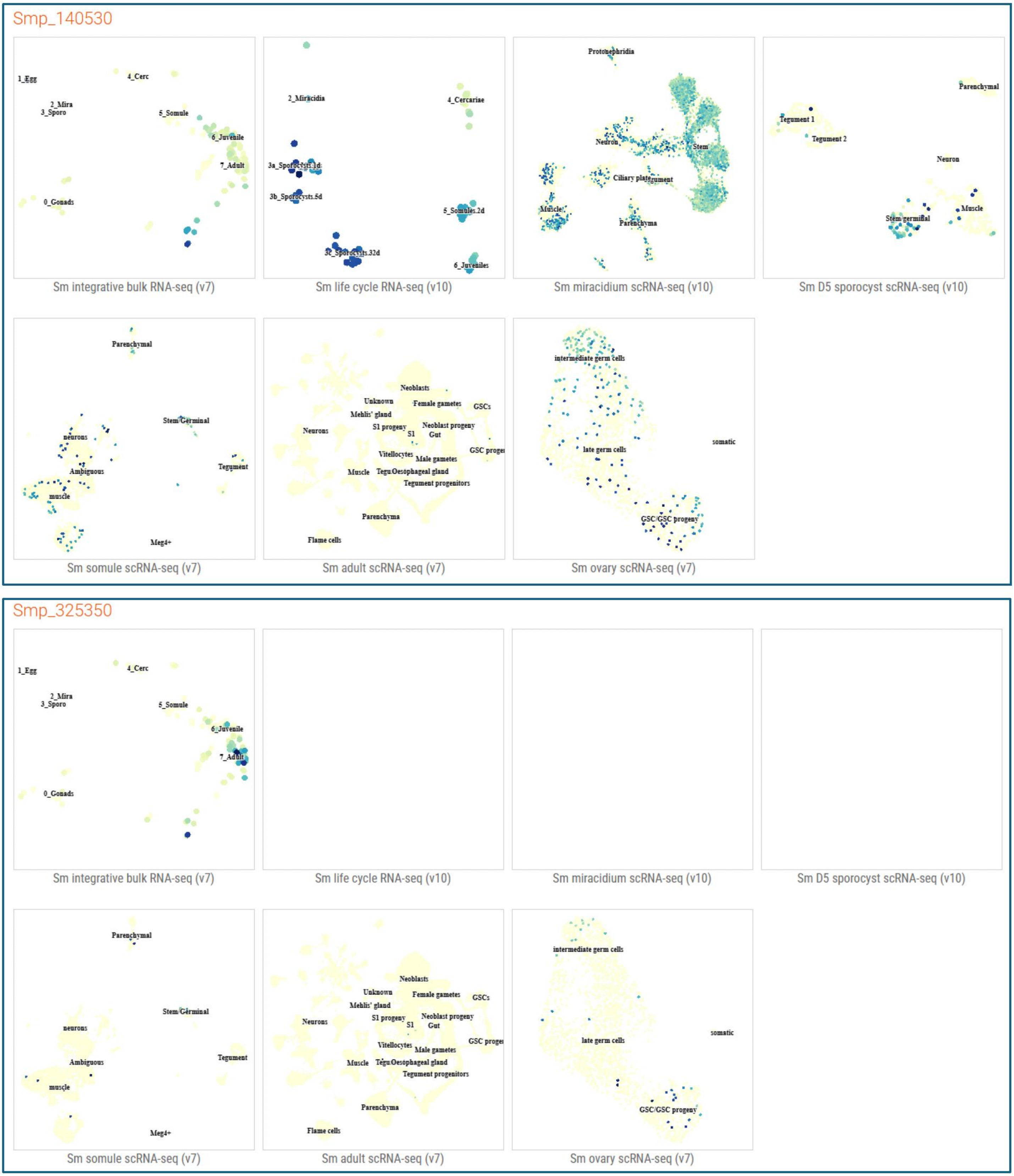
Smp_140530
1_Egg
4_Cerc
2_Mira
3_Sporo
5_Somule
6_Juvenile
7_Adult
0_Gonads
Sm integrative bulk RNA-seq (v7)
2_Miracidia
4_Cercariae
3a_Sporocysts.1d
3b_Sporocysts.5d
5_Somules.2d
3c_Sporocysts.32d
6_Juveniles
Sm life cycle RNA-seq (v10)
Protonephridia
Neuron
Stem
Ciliary plate
Tegument
Muscle
Parenchyma
Sm miracidium scRNA-seq (v10)
Parenchymal
Tegument 1
Tegument 2
Neuron
Muscle
Stem germinal
Sm D5 sporocyst scRNA-seq (v10)
Parenchymal
Stem/Germinal
neurons
Ambiguous
Tegument
muscle
Meg4+
Sm somule scRNA-seq (v7)
Neoblasts
Unknown
Female gametes
GSCs
Mehlis' gland
S1 progeny
Neoblast progeny
Neurons
S1
Gut
Vitellocytes
Male gametes
GSC proge
Muscle
Tegu
Oesophageal gland
Tegument progenitors
Parenchyma
Flame cells
Sm adult scRNA-seq (v7)
intermediate germ cells
somatic
late germ cells
GSC/GSC progeny
Sm ovary scRNA-seq (v7)
Smp_325350
1_Egg
4_Cerc
2_Mira
3_Sporo
5_Somule
6_Juvenile
7_Adult
0_Gonads
Sm integrative bulk RNA-seq (v7)
Sm life cycle RNA-seq (v10)
Sm miracidium scRNA-seq (v10)
Sm D5 sporocyst scRNA-seq (v10)
Parenchymal
Stem/Germinal
neurons
Ambiguous
Tegument
muscle
Meg4+
Sm somule scRNA-seq (v7)
Neoblasts
Unknown
Female gametes
GSCs
Mehlis' gland
S1 progeny
Neoblast progeny
Neurons
S1
Gut
Vitellocytes
Male gametes
GSC proge
Muscle
Tegu
Oesophageal gland
Tegument progenitors
Parenchyma
Flame cells
Sm adult scRNA-seq (v7)
intermediate germ cells
somatic
late germ cells
GSC/GSC progeny
Sm ovary scRNA-seq (v7)

Figure S11
